# In Vitro Inhibitory Activity of Corilagin and Punicalagin Against *Toxoplasma gondii* and Their Mechanism(s) of Action

**DOI:** 10.3390/antibiotics14040336

**Published:** 2025-03-24

**Authors:** Nicole T. Green-Ross, Homa Nath Sharma, Audrey Napier, Boakai K. Robertson, Robert L. Green, Daniel A. Abugri

**Affiliations:** 1 Department of Biological Sciences, Alabama State University, Montgomery, AL 36104, USA; nicole.t.ross2.mil@health.mil (N.T.G.-R.); hsharma1952@myasu.alasu.edu (H.N.S.); anapier@alasu.edu (A.N.); brobertson@alasu.edu (B.K.R.); 2 Microbiology Ph.D. Program, Alabama State University, Montgomery, AL 36104, USA; 3 Laboratory of Ethnomedicine, Parasitology and Drug Discovery, Alabama State University, Montgomery, AL 36104, USA; 4 Department of Physical Sciences, College of Science, Technology, Engineering and Mathematics, Alabama State University, Montgomery, AL 36104, USA; rgreen@alasu.edu

**Keywords:** corilagin, punicalagin, pyrimethamine, inhibit, *T. gondii*

## Abstract

**Background/Objectives:** Toxoplasmosis is a zoonotic disease caused by *Toxoplasma gondii*. The parasite infection in humans continues to rise due to an increasing seroprevalence rate in domestic and wild warm-blooded animals that serve as a major reservoir of the parasite. There are fewer drugs available for the treatment of toxoplasmosis. However, these drugs are limited in efficacy against tachyzoites and bradyzoites. Also, there are clinical side effects and geographical barriers to their use, especially in immunocompromised patients, children, and pregnant women. Tannins, a class of natural products, are known to have antimicrobial properties. However, little is known about the effects of Corilagin (CG) and Punicalagin (PU), which are classified as tannins, on *T. gondii* growth and their possible mechanism of action in vitro. We hypothesize that CG and PU could inhibit *T. gondii* growth in vitro and cause mitochondria membrane disruption via oxidative stress. **Methods:** Here, we investigated the anti-*T. gondii* activity of the two named tannins using a fluorescent-based reporter assay. **Results:** The 50% effective concentrations (EC_50s_) values for CG and PU that inhibited *T. gondii* parasites growth in vitro were determined to be 3.09 and 19.33 µM, respectively. Pyrimethamine (PY) was used as a standard control which gave an EC_50_ value of 0.25 µM. Interestingly, CG and PU were observed to cause high reactive oxygen species (ROS) and mitochondrial superoxide (MitoSOX) production in tachyzoites. This resulted in a strong mitochondria membrane potential (MMP) disruption in *T. gondii* tachyzoites. **Conclusions:** Therefore, the possible mechanism(s) of action of CG and PU against *T. gondii* is associated with the disruption of the mitochondria redox biology. Thus, the high ROS and MitoSOX produced as a result of these compounds created high oxidative stress, leading to mitochondrial dysfunction.

## 1. Introduction

*Toxoplasma gondii* (*T. gondii*) is a global cosmopolitan parasite with great socio-economic, veterinary, and public health issues and causes the disease toxoplasmosis. Currently, Pyrimethamine (PY) and sulfadiazine (SZ) combination have been the golden standard for the treatment of *T. gondii* infection [1,2]. However, studies have shown that these combinations, along with other drugs like spiramycin and atovaquone, have serious clinical effects such as toxicity in pregnant women and their fetuses, immunosuppressed individuals (HIV–AIDS), cancer patients, and organ transplant recipients [3,4,5,6]. Furthermore, it has been recorded that pyrimethamine plus sulfadiazine, atovaquone, among other drugs, do exhibit treatment failure in tachyzoites and have not even been effective against their bradyzoite forms [4,7]. Thus, there is an urgent necessity to identify and develop new anti-*Toxoplasma gondii* inhibitors.

Natural products are known to be a useful source of new medicines against pathogens. For example, steroids extracted from *Trametes versicolor* and other mushrooms have inhibited leishmania [8,9,10], 3-deoxyanthocyanidins derived from *Sorghum bicolor* leaves and tannin-based compounds against *Toxoplasma gondii* growth [11,12,13], as well as phenolic and flavonoids against *Toxoplasma gondii* growth [14].

Corilagin (CG), is a natural polyphenol and a member of the tannin family that was originally isolated from *Arctostaphylos uvaursi* [15,16]. CG, a gallotannin, is one of the most active components in medicinal plants. This compound has displayed cell arrest and activated mitochondria and endoplasmic reticulum stress pathways in cancer cells [17,18]. Furthermore, CG has been found to have antihypertensive [19], antibacterial [20,21], and antiviral properties [22].

Another natural product, Punicalagin (PU), is an ellagitannin, known to be in abundance in pomegranate peels, and has been reported to exhibit pharmacological and biological properties such as antioxidant, antimicrobial [23], antiviral [24,25], anti-obesity [26], anticancer, anti-inflammatory, and immunosuppressive activities [27,28]. More specifically, PU has been reported to inhibit and block herpes simplex virus type 1 (HSV-1) entry and spread. In addition, PU has inhibited bacteria (*Staphylococcus aureus*) [23] and has also been known to target proteins that support the SARS-CoV-2 virus life cycle [28].

Although CG and PU have been known for their antibacterial, antiviral, anticancer, and anti-obesity properties, little is known about these compounds’ effect on *T. gondii* tachyzoites’ growth and their possible mechanism of action. Compounds with highly rich polyphenols have been reported to perturb the mitochondria membrane potential and result in reactive oxygen species production [29,30]. Mitochondria are an important organelle for energy biogenesis in cells. Based on this insight, it is well-known that *T. gondii* has a single mitochondrion and, therefore, finding compounds that could target the mitochondria directly or indirectly through oxidative stress pathways is important for the development of new effective drugs against the parasite growth. We hypothesized that CG and PU inhibit *T. gondii* growth by disrupting mitochondrial function via ROS generation. Thus, this study was carried out to assess CG and PU inhibitory activity, cytotoxicity, reactive oxygen species (ROS) production, mitochondria superoxide production, and mitochondria membrane potential defects in vitro.

## 2. Results

### 2.1. In Vitro Inhibition of T. gondii Growth and Cytotoxicity

In this study, we report for the first time anti-*T. gondii* (RH-RFP) tachyzoites inhibitory activity of CG and PU tested in vitro. The EC_50s_ values for CG and PU against *T. gondii* growth in vitro was calculated to be 3.09 (1.86 to 5.06) µM and 19.33 (13.13 to 28.75) µM for parasites for parasites, respectively (Table 1). The CC_50s_ values for CG and PU were determined to be 37.27 µM and 21.30 for hTERT cells, respectively (Table 1). PY was concomitantly tested with CG and PU, which gave an EC_50_ value of 0.25 (0.12 to 0.43) µM. The selectivity index (SI) values for CG were calculated to be 12.20, whilst that of PU was determined to be 1.10.

To explore the possible mechanism(s) of action of CG and PU against *T. gondii* tachyzoites growth in vitro, we performed a redox biology assay which included mitochondria superoxide and reactive oxygen species (ROS) production, as well as mitochondria membrane potential function.

### 2.2. Effect of CG and PU on Mitochondria Superoxide (MitoSOX) and Reactive Oxygen Species (ROS) Production in T. gondii Tachyzoites

**Figure 1:**
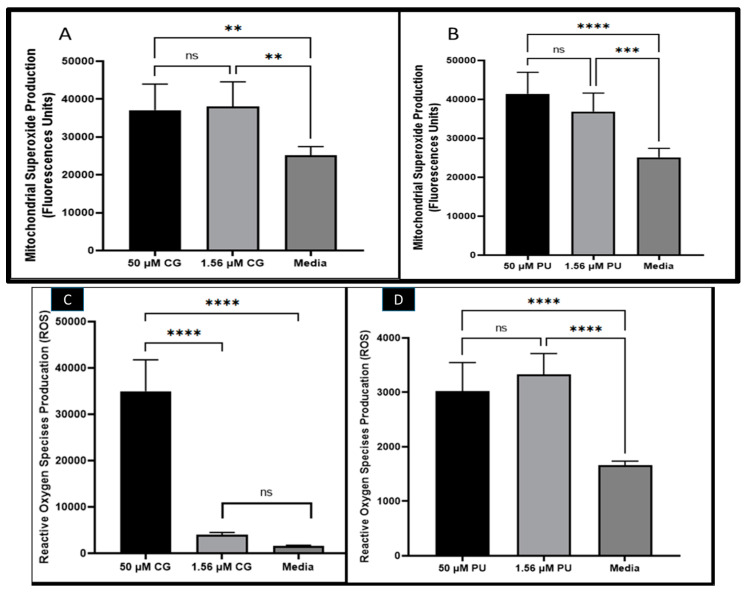
(**A**) CG and (**B**) PU effects on mitochondrial superoxide production (MitoSOX) in *T. gondii* tachyzoites. (**C**) CG and (**D**) PU effects on reactive oxygen species production (ROS) in *T. gondii* tachyzoites. **, ***, **** represent means plus standard deviation of six independent experiments performed in single wells with *p* < 0.01, *p* < 0.001 and *p* < 0.0001. ns—not significant differences.

It was observed that CG and PU induced in *T. gondii* tachyzoites mitochondria superoxide production (Figure 1A,B). There were statistically significant differences in MitoSOX production between the selected concentrations (1.56 and 50 µM) and that of the media, which served as a negative control at *p* < 0.001. In contrast, there were no statistical differences between 1.56 µM of CG-treated tachyzoites and that of the 50 µM of CG- and PU-treated parasites, respectively.

In the ROS assessment studies with CG and PU, we observed that CG and PU significantly induced in *T. gondii* tachyzoites ROS production in vitro (Figure 1C,D). Interestingly, it was observed that the ROS production in tachyzoites induced by CG was statistically significant between 50 µM and 1.56 µM of CG tested. A similar trend was observed between 50 µM of CG and the media (control) with *p* < 0.0001. Contrarily, there was no significant difference between the media and 1.56 µM of CG tested (Figure 1C). In the PUROS data, there was a significant difference in ROS production between the 50 µM treatment and the media (negative control) (*p* < 0.0001). Similarly, the ROS produced when 1.56 µM of PU was tested on extracellular parasites compared to the media (negative control) treated group was statistically different with *p* < 0.0001. No statistical difference was observed between the two concentrations of PU tested (Figure 1D).

### 2.3. Mitochondrial Membrane Potential Disruption

**Figure 2:**
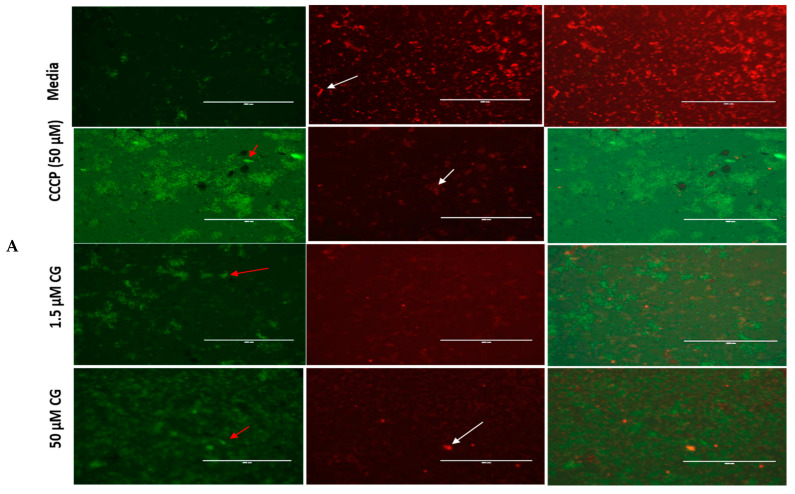

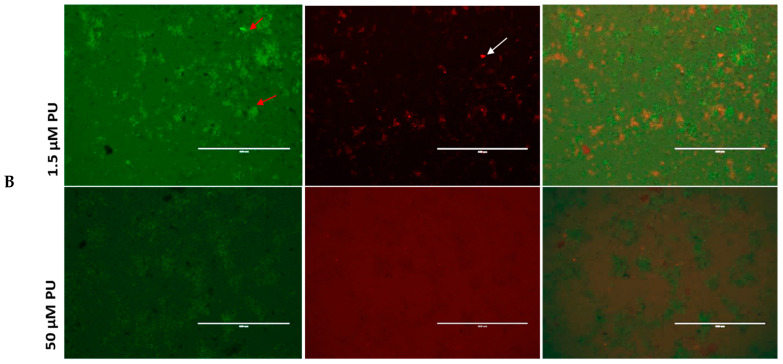
(**A**) CG and (**B**) PU disrupt mitochondria membrane potential (MMP) in *T. gondii* tachyzoites. The red and white arrows indicate parasites. The red sparking spots (parasites) indicate parasites whose mitochondrial membranes are intact (JC-1 aggregates) while the green spots indicate parasites whose mitochondrial membranes have been compromised (JC-1 monomers) by the CG and PU treatment. Scale bar = 400 µm using a 20× objective lens. The Carbonyl cyanide m-chlorophenyl hydrazone (CCCP, from Alfa Aesar, Haverhill, MA, USA) was used as a positive control; it is known to cause mitochondrial membrane disruption by reducing the JC-1 aggregates and increasing the JC-1 Monomers in cells.

It is well-known that the mitochondrion is an organelle whose primary function is to synthesize ATP for cellular use. Also, this organelle supports cellular respiration, fatty acid degradation, nuclear iron–sulfur protein synthesis, tRNA modification, DNA synthesis and repair, and protein translation. Using a red fluorescent probe JC-1 mitochondria test kit, we showed that CG and PU treatment on *T. gondii* tachyzoites caused alteration of *T. gondii* mitochondrial membrane potential (Figure 2A,B). The results were consistent with our standard positive control (Carbonyl cyanide m-chlorophenyl hydrazone (CCCP, from Alfa Aesar, Haverhill, MA, USA)) which is known to cause mitochondrial membrane disruption by penetrating through the membrane, causing the reduction of the JC-1 aggregates and increasing of the JC-1 Monomers in the cells.

### 2.4. Integrity of Host Cells with Parasites and Drugs Interaction During Growth Inhibition Assay

**Figure 3:**
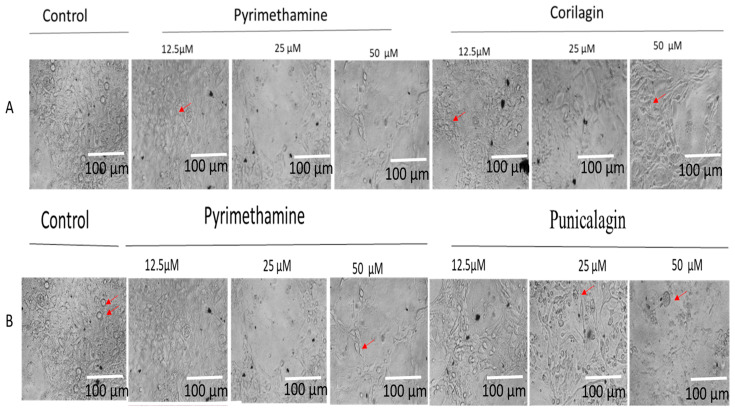
(**A**) CG and (**B**) PU indicate the integrity of host cells under parasite infection and drug treatment at different concentrations (12.5, 25, and 50 µM) at 72 h post-infection using pyrimethamine as positive control and growth media (10%FBS and 1% PSF). The red arrows indicate parasite-infected cells. Scale bar of 100 µm with 20× objective lens using Zeiss Axiovert 100 inverted phase contrast microscope (Zeiss, Oberkochen, Germany).

There was not any disruption of host cells at different concentrations of PY, CG, and PU treatment of tachyzoites following 72-h interactions (Figure 3A,B), respectively. From the phase-contrast microscopy, it was evident that parasite infections were reduced in a concentration-dependent manner in CG, PU, and PY treatment at 12.5 to 50 µM compared to the negative control (media with only parasites and host cells).

## 3. Discussion

*T. gondii* infections in companion and food animals are on the increase [31,32]. Currently, the antiparasitic agents used for the treatment of toxoplasmosis are limited clinically and geographically, thus creating a huge challenge to the control or treatment of the different stages of the parasites. To overcome this, several ongoing studies are using natural and synthetic compounds to decipher their inhibitory, cytotoxicity, and mechanism of action against the parasite in vitro and in vivo [16,20,33,34]. However, these important compounds have not been properly verified and clinically tested as being effective in humans, thus calling for further testing of compound libraries for future in vivo testing and chemical modification.

In this work, we discovered that CG was highly effective against *T. gondii* tachyzoite growth (Table 1) following 72-h interaction with minimum cytotoxic concentrations. Also, PU was effective but not comparable to CG with an inhibitory EC_50_ value (Table 1). The selectivity index (SI) value for CG was 12. This implies that CG inhibitory activity against parasites was 12 times greater than its cytotoxic effect on hTERT cells tested at 72 h and will have a broader spectrum in killing *T. gondii* tachyzoites when confirmed to be effective and safe in vivo. Also, our results showed that the selected compounds did not damage the host cells’ integrity (Figure 3A,B). The CG has been previously reported to have anti-bacterial [21], and anti-viral properties [22]. The EC_50s_ values obtained were comparable to standard drugs (e.g., PY)—with values of 0.1 to 3 µM [30,35]—and other potent natural compounds like quercetin, taxifolin, 3-deoxyanthocyandins [36,37,38], and synthetic compounds such as N-(pyridin-2-yl)-4-(pyridine-2-yl) thiazol-2-amine) tested against the parasite [39], styryl 4-oxo-1,3-benzoxazin-4-one KG3, tetrahydrobenzo[b]pyran KG7, and benzoquinone hydrazone KG8 [40]. The limitation of our study is that the EC_50s_ were not time-dependent. Furthermore, these compounds were not tested with current drugs to determine their synergistic effects. Although the PY was more effective than the CG and PU, we believe that combining these compounds with Atovaquone and other compounds that target the mitochondrial membrane could make them synergistic and active against parasite proliferation [29]. This assumption requires further studies. Additionally, these compounds have high antioxidant properties and are abundantly found in pomegranate fruits; they could act as a booster to the immune system of infected individuals to overcome the parasite’s survival via oxidative stress and eventually lead to the parasite’s death.

To explore the mechanism of action associated with CG- and PU-inhibitory activity against *T. gondii* in vitro. We performed several biochemical assays such as mitochondria superoxide (MitoSOX) production, reactive oxygen species (ROS) generation, and mitochondria membrane potential (MMP).

ROS production in cells has been associated with cellular imbalance. Therefore, our results of high production of ROS could lead to high production of free radicals, lipid radicals, and aldehydes (toxic) compounds that cause damage to the host and pathogens’ membranes and mitochondria [30,33]. We believe that such excessive ROS productions could cause stress in parasites, resulting in apoptosis-mediated pathway disruptions and eventually cell death. This has been reported in Leishmania, *Toxoplasma gondii,* and in *Plasmodium* spp. parasites [41,42]. It is not clear why the CG requires higher concentration to exert high ROS production than that of PU. However, we believe that the observation could be due to the structural difference and the presence of the hydroxyl (OH) groups, their availability, and their intracellular reactive capabilities to elicit reactive oxygen species generation. Thus, future studies will be required to examine these speculations. The non-statistical difference observed from the 50 and the 1.56 µM of PU could imply that the ROS generation is not dose-dependent. However, since there were only two concentrations tested with the current findings, we will require future investigation with serially diluted concentrations to confirm or disprove this conjecture.

CG and PU were observed to trigger mitochondrial superoxide productions in tachyzoites. This cellular production of the superoxide could have caused both host and parasite cells to have imbalanced homeostasis, eventually leading to parasite death. These conjectures have been reported in eukaryotic cells [34,41,43].

Also of interest was the fact that the increase in ROS and MitoSOX productions resulted in the mitochondria membrane being compromised. Notably, CG and PU were observed to cause remarkable mitochondrial membrane potential disruption and thus could affect the single mitochondria found in *T. gondii*. This implies that the mitochondria of the tachyzoite might be dysfunctional, resulting in parasites not meeting their energy expenditure and eventually their death. Generally, it is well-documented that *T. gondii*’s ability to attach, invade, and replicate intracellularly is heavily energy-dependent. Our findings concurred with other researchers that have published natural and synthetic compounds that target mitochondria membrane and lead to depolarization in parasites and eventually parasite death [29,30,34,41,44,45].

Previous studies on esophagus cancer showed that CG induces apoptosis and cell cycle arrest [46]. The mechanism of action of CG has been associated with the activation of the mitochondria and endoplasmic reticulum-related apoptosis signaling pathways [46]. The mitochondrion is a site for lipid synthesis, transcription, and translational processes. Also, in *T. gondii*, the intracellular parasite requires energy for survival, invasion, and proliferation. Thus, disrupting the mitochondria, which is one way the parasite generates its energy, is likely to shut its glycolysis pathways and eventually cause an imbalance between the mitochondria and glycolysis activity. This may eventually lead to parasite death [47]. Kakiuchi et al. [48] have reported that CG inhibits reverse transcriptase activity and thus support our observation that the mitochondria dysfunction observed could affect the transcriptional and translation processes in the tachyzoites. PU has also been reported to stop viral entry [24,25] and inhibit bacteria growth. Therefore, PU might have blocked *T. gondii* tachyzoites adhesion, entry, and proliferation.

**Figure 4:**
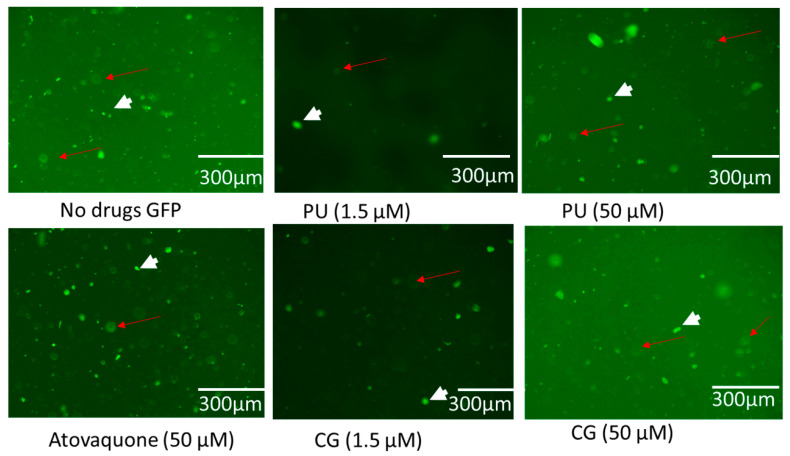
Showing the effect of PU, CG, and ATOV treatment on intracellular parasites and host cells at different concentrations (1.5 and 50 µM) at 72 h post-infection. No drug (0 µM). The short arrows represent parasites while red arrows represent host cells labelled with cell light mitochondria GFP.

However, the compounds (CG and PU) have been observed to inhibit parasites and cause potential disruption of the mitochondrial membrane. It has been reported that these compounds do not induce mitochondria impairment and oxidative stress in host cells [20,46]. We observed that intracellular parasites and HFF cells labelled with cell light mitochondria GFP dye showed brighter parasites in GFP than in host cells (Figure 4). This confirmed another finding reported in [46]. Furthermore, it inferred that both compounds may not have direct effect on the mitochondria of host cells.

## 4. Materials and Methods

### 4.1. Chemicals Acquisition

Corilagin, Punicalagin, and Pyrimethamine were obtained from Santa Cruz Biotechnology Inc., Dallas, TX, USA. Non-phenol red 0.25 Trypsin EDTA solution %1 PSG (Penicillin–Streptomycin–Glutamate), 5% Bovine Calf Serum (BSC), non-phenol red Dulbecco’s Modified Eagle Medium (Thermo Fisher Scientific, Waltham, CA, USA). Compounds (Corilagin and Punicalagin) and pyrimethamine were prepared in DMSO and serially diluted using growth media, with DMSO percentage in each concentration to be 0.1%, and stored at −20 °C before the test. Each stock was serially diluted using a clear media (non-phenol red) growth media. Concentrations were prepared from 0 to 50 µM.

### 4.2. Maintenance of Parasites and Cell Cultures

Human Telomerase Reverse Transcriptase (hTERT) immortalized Human Foreskin Fibroblast (HFF) cells were obtained from Professor Silva N. J. Moreno and maintained using Dulbecco’s Modified Eagle’s Medium (DMEM) supplemented with 5% (*v/v*) fetal bovine serum (FBS), 200 nM L-glutamine, and 1% (*v/v*) penicillin–streptomycin obtained from (Life Technologies, St. Louis, MO, USA) and incubated with 5% CO_2_ and 95% air at 37 °C. *T. gondii* (Type I strain, RH-RFP expressing red fluorescent protein) in culture. We initially seeded hTERT HFF cells into 96-well plates using the volume of 200 µL or into 12-well plates using the volume of 2 mL of DMEM medium and allowed them to grow to 80–100% before use. *T. gondii* tachyzoites were harvested by passing them through a 27-gauge needle followed by filtering through a 3 µm filter.

### 4.3. Cytotoxicity

A group of 1.24 × 10^6^ hTERT cells/200 μL/well were seeded into the flat black bottom 96-well plates and incubated at 37 °C with 5% CO_2_ for 24 h [30]. Then dead cells were removed by washing three times with 1× Phosphate Buffered Saline (1× PBS). The wells were refilled with 100 μL of media and 100 μL of CG, and PU were prepared in serially diluted concentrations of 0, 0.78, 1.53, 3.06, 6.125, 12.5, 25, and 50 μM. Pyrimethamine was used as a positive control with the same concentration ranges. The plates were incubated for 72 h, then 10 μL of Alamar Blue (Abcam, Waltham, MA, USA) was added into the plates and incubated for an hour at 37 °C with 5% CO_2_. The fluorescence intensities were read at 485/535 nm. The percentage of cell viability was calculated using the formula stated in Huffman et al. [30]. The CC_50s_ values were determined using Graph Pad Prism software. Experiments were performed in triplicate (n = 3).

### 4.4. Growth Inhibition Assays

To assess the proliferation inhibition, approximately 4 × 10^4^ hTERT cells/200 μL volumes were added to black bottom 96-well plates and incubated at 37 °C with 5% CO_2_ for 24 h. The wells were washed with 1× PBS three times and 1 × 10^3^ /100 μL tachyzoites of *T. gondii* RH-RFP type I strain was added and incubated at 5% CO_2_ and 95% for three hours. CG and PU (0, 0.78, 1.56, 3.12, 6.12, 12.5, 25, and 50 μM) with the same concentration ranges for pyrimethamine were added to their designated wells. The plates were incubated at 37 °C with 5% CO_2_ and were read for 72 h using a Tecan 200 F infinite microplate reader (Tecan Austria GmbH, Grodig, Austria) with excitation set at 560 nm and emission at 630 nm. The percentage effective inhibition concentration (EC_50s_) was calculated by normalizing the control to zero and the highest concentrations to 100%.

### 4.5. Reactive Oxidative Species (ROS) Production

CG and PU are highly antioxidant. Thus, to find out the possible mechanism of action of CG, extracellular parasites (with a concentration of 1 × 10^5^ tachyzoites/100 µL) of *T. gondii* RH-W (wild-type) strain were added to flat black bottom 96-well plates. Then, CG and PU at concentrations of 50 µM and 1.56 µM were added to the parasites in designated wells. Reaction plates were incubated at 37 °C with 5% CO_2_ and 95% atmospheric air for 30 min. Exactly 10 µL of 5 µM oxidative stress-orange reagent (CellROX™ Orange, (Life Technologies Corporation, St. Louis, MA, USA) was added to each well and covered with aluminum foil, and kept in the incubator at 37 °C for 15 min according to the CellROX™ Orange reagent (C10443) protocol provided by Invitrogen, USA. Next, the wells’ fluorescence intensities were measured using a Tecan 200 F infinite microplate reader (Tecan Austria GmbH, Grodig, Austria) with excitation/emission set at 560/635 nm [29,30].

### 4.6. Mitochondrial Superoxide Production Assay

To evaluate whether CG induces superoxide production, extracellular parasites (1 × 10^5^ tachyzoites/100 μL) of *T. gondii* RH-W (wild-type) strain were added to black 96-well plates. CG and PU with concentrations of 1.56 and 50 μM were added to their designated wells, and the media serving as a negative control. Black plates containing parasites and treatments were incubated at 37 °C with 5% CO_2_ for 3 h. Next, 50 μL of 5 μM of MitoSOX^TM^ reagent was added, and the plates were covered with aluminum foil, and the plates were incubated at 37 °C for 30 min. Plates were measured at excitation 485 nm and emission at 535 nm using a Tecan 200 F infinite microplate reader (Tecan Austria GmbH, Grodig, Austria). Experiments were performed in triplicate (n = 3) [29].

### 4.7. CG and PU Effect on T. gondii Mitochondrial Membrane Potential

To determine whether the high superoxide production and reactive oxygen species generated by CG and PU could have any effect on *T. gondii* tachyzoites’ mitochondrial function, we used the cationic probe JC-1 (Thermo Fisher Scientific, Waltham, CA, USA) test kit as presented in our previous works [29,30]. Approximately 1 × 10^5^ purified tachyzoites from *T. gondii* RH wild-type strain/50 μL media were added to a designated black 96-well plate (Costar, Corning Inc., Corning, NY, USA). Afterwards, 50 μL of 1.56 and 50 μM of CG and PU were added to the designated wells, while 50 μM of Carbonyl cyanide m-chlorophenyl hydrazone (CCCP, from Alfa Aesar, Haverhill, MA, USA) was used as a positive control and assay buffer (AB) as a negative control; the wells were incubated for 8 h at 37 °C with 5% CO_2_ [29,30]. At the 8 h incubation period, 10 μL of JC-1 dye was added to the plates and incubated for 45 min in the dark. The plates were centrifuged at 12 °C for 2000 rpm for 5 min. The supernatant was carefully removed, and parasites were suspended in a 100 μL assay buffer, followed by centrifugation and removal of the supernatant. Parasites were suspended in 100 μL of the assay buffer and their fluorescence was measured at 560/635 nm (JC-1 J-aggregates) and at 485/535 nm (JC-1, J-monomers) using a Tecan 200 F infinite microplate reader. The ratio of fluorescence values of J-aggregates to monomers was determined according to Huffman et al. [29,30]. Images were captured using an EVOS FL fluorescence microscope (Invitrogen Life Technologies, Carlsbad, CA, USA).

### 4.8. Effect of PU, CG and AT on Intracellar T. gondii Tachyzoites and HFF Host Cells

Here, 4.0 × 10^5^ HFF cells were cultured in black 96-well plate and grown to confluency. The media was changed with new growth media (2.5% of FBS/1% PSF) and 2.37 × 10^4^ parasites /50 µL FOU strain that does not have a fluorescent reporter were added to the wells. After 10 min, drugs (PU, CG and atovaquone (AT)) were added at 1.56 and 50 µM and incubated at 37 °C with 5% CO_2_ for 72 h, after which the CellLight Mitochondria-GFP, BacMam 2.0, (Life Technologies Corporation, St. Louis, MA, USA) (2 µL) that labels mitochondria with green fluorescent protein (GFP) in live cells was added. Plates were incubated overnight under standard culture conditions and the cells were imaged using an EVOS FL fluorescence microscope (Invitrogen Life Technologies, Carlsbad, CA, USA).

### 4.9. Statistical Analysis

The CC_50s_ and EC_50s_ values were determined using Graph Pad Prism 9.2 software. The statistical differences in each compound’s effect on mitochondria membrane potential, reactive oxygen species, and mitochondria superoxide production were compared to the control groups using one-way ANOVA. Means with statistical significance differences were defined by *p* values less than 0.05.

## 5. Conclusions

In summary, our results showed that CG and PU are effective inhibitors of *T. gondii* tachyzoites growth in vitro. Also, the possible mechanism(s) of action of CG and PU against *T. gondii* growth is through perturbation of the mitochondria membrane via excessive production of ROS and MitoSOX, which are associated with apoptosis-mediated enzymes, leading to parasite death. However, PU was slightly toxic to host cells and thus required further chemical modifications for future in vitro and in vivo testing. Overall, future studies will be required to test CG and PU in vivo to validate their parasitic clearances and safety for future development of anti-toxoplasma drugs.

## Figures and Tables

**Table 1:** A 50% effective concentration (EC_50_) inhibition of *T. gondii* growth using PY, CG, and PU following 72-h interaction in vitro.

Compounds (µM)	EC_50s_ with 95% CL	CC_50s_	SI Value
PY	0.25 (0.12 to 0.43)	Nd	Nd
CG	3.09 (1.86 to 5.06)	37.27	12.20
PU	19.33 (13.13 to 28.75)	21.30	1.10

Results are presented as means with 95% confidence level (CL) of six replicates. EC_50_—50% effective concentration of inhibition of *T. gondii* tachyzoites. CC_50_—cytotoxic concentration that inhibits 50% host cells. SI—is a selective index (CC_50_/EC_50_). Nd—not determined.

## Data Availability

The original contributions presented in this study are included in the article. Further inquiries can be directed to the corresponding author.
